# Ultrasound-Assisted Extraction, Chemical Characterization, and Impact on Cell Viability of Food Wastes Derived from Southern Italy Autochthonous Citrus Fruits

**DOI:** 10.3390/antiox11020285

**Published:** 2022-01-30

**Authors:** Gabriele Carullo, Anna Ramunno, Eduardo Maria Sommella, Michele De Luca, Emilia Lucia Belsito, Luca Frattaruolo, Matteo Brindisi, Pietro Campiglia, Anna Rita Cappello, Francesca Aiello

**Affiliations:** 1 Department of Biotechnology, Chemistry and Pharmacy, University of Siena, Via Aldo Moro 2, 53100 Siena, Italy; gabriele.carullo@unisi.it; 2 Department of Pharmacy, University of Salerno, Via G. Paolo II 132, 84084 Fisciano, Italy; aramunno@unisa.it (A.R.); esommella@unisa.it (E.M.S.); pcampiglia@unisa.it (P.C.); 3 Department of Pharmacy, Health and Nutritional Sciences, University of Calabria, Edificio Polifunzionale, 87036 Arcavacata di Rende, Italy; michele.deluca@unical.it (M.D.L.); emilialucia.belsito@unical.it (E.L.B.); luca.frattaruolo@unical.it (L.F.); matteo.brindisi@unical.it (M.B.); annarita.cappello@unical.it (A.R.C.)

**Keywords:** antioxidant activity, antiproliferative effects, food wastes, citrus fruits

## Abstract

Citrus fruits are one of the principal fruits used to produce juices. Over the years, these fruits have been recognized as new health-promoting agents. In this work, food wastes derived from autochthonous citrus fruits of Southern Italy, named Limone di Rocca Imperiale, Arancia Rossa Moro, and Arancia Bionda Tardivo from Trebisacce, were analyzed. After fresh-squeezing juice, peel and pomace were employed to obtain six different extracts using an ultrasound-assisted method in a hydroalcoholic solvent. The extracts were analyzed in terms of qualitative composition, antioxidant properties, and antiproliferative activity on MCF-7, MDA-MB-231, and BJ-hTERT cell lines. GC-MS and LC-ESI-MS analyses showed different compounds: of note, limonin-hexoside, neodiosmin, obacunone glucoside, and diacetyl nomilinic acid glucoside have been identified as limonoid structures present in all the samples, in addition to different polyphenols including naringenin-glucoside, hesperetin-*O*-hexoside-*O*-rhamnoside-*O*-glucoside, diferuloyl-glucaric acid ester, chlorogenic acid, and the presence of fatty acids such as palmitic, myristic, and linoleic acids. These extracts were able to exert antioxidant activity as demonstrated by DPPH and ABTS assays and, although at higher doses, to reduce the cell viability of different solid tumor cell lines, as shown in MTT assays.

## 1. Introduction

Citrus fruits comprise orange, red-orange, bitter orange, lemon, lime, bergamot, and grapefruit. These fruits have a thick cortex that contains large amounts of aromatic oils, well known for their cosmeceutical use (e.g., production of *Original Eau de Cologne*) [1]. Citrus fruits are a rich source of biocomponents including flavonoids, dietary fibers (DFs), essential oils (EOs), synephrines, limonoids, and carotenoids. These functional components are well-known health-promoting agents, with reported activity against cardiovascular diseases, inflammatory conditions, viral infections, and cancer diseases [2,3,4,5,6]. Among the most representative bioactives, flavanones are the most abundant polyphenols present with reported biological activities. These include radical-scavenging, anti-inflammatory, anti-mutagenic, vasorelaxant, and vasoprotective properties [7,8,9,10]. The most intriguing class of compounds is represented by limonoids, considered selective tetranortriterpenoid compounds present in citrus fruits as reported in the literature [11]. Limonoids are abundant in peels and seeds and exert different pharmacological activities including anticancer, antimicrobial, antioxidant, antidiabetic, and insecticidal, among others [12,13]. One of the most studied, limonin, displayed good antiproliferative activity in the micromolar range, while its derivatives showed interesting anti-inflammatory and anti-nociceptive properties [14]. In particular, limonin demonstrated cytotoxic effects in a p53-dependent signaling network involving the phosphorylations of ERK, p38, and both serine residues (Ser 468 and Ser 536) of NFκB pathways as found in MCF-7 breast cancer cells, but not in p53 mutant MDA-MB-231 cells [15]. Obacunone (**2**) is an α,β-unsaturated-ε-lactone, known for its larvicidal activity [12]. Regarding antitumor activity, **2** could reduce the development of colonic adenocarcinoma in the F344 rat model. In SH-SY5Y neuroblastoma cells, it was able to increase caspase 3/7 activity, suggesting an apoptosis-inducing effect in a concentration- and time-dependent manner [15]. Limonoids of different nature were also combined with curcumin in SW480 colorectal cancer cells. Results of cell proliferation assays suggest that combinations of limonoids with curcumin increase total cellular caspase-3 activity and Bax/Bcl-2 ratio [16]. These compounds, together with others, are abundant in peels and also in pomace, which are generally industrial wastes after juice production [17]. Over the years, citrus wastes have been appreciated and reused to achieve new essential oils useful in cosmetics, pharmaceuticals, and other industrial and domestic applications [18]. Furthermore, wastes were used to extract pectins to combine them with other substances to develop novel upgraded biocomposites for mulching applications [19]. In the food industry, citrus wastes have been used as new additives, which showed sourness, bitterness, and orange-like taste, and overall acceptance similar to commercially citrus-flavored drinks [20]. In this field, it has been shown that citrus wastes (particularly peels and pomaces) are intriguing sources of limonoids and fatty acids [21]. Nevertheless, the literature did not report concise information about the three different types of citrus from southern Italy, that are “Limone di Rocca Imperiale”, “Arancia Rossa Moro”, and “Arancia Bionda di Trebisacce”.

The Protected Geographical Indication (PGI) Limone di Rocca Imperiale is reserved for fruits from cultivars of the Feminello group, belonging to the botanical species *Citrus limon* Burm, known in the area with the name Limone di Rocca Imperiale. When released for consumption, the fruits of Limone di Rocca Imperiale have the following characteristics: peel color, from light green to yellow; fruit shape, from elliptical elongated to spheroidal; dimensions, from medium to large, with a caliber not less than 53 mm; and weight not less than 100 g. The production area of the Limone di Rocca Imperiale PGI coincides with the administrative territory of the municipality of Rocca Imperiale (CS-Italy). The Arancia Rossa Moro, *Citrus sinensis* var. Moro, has a globular or ovoid shape, orange peel with more intense shades on one side of the fruit, and an intense vinous red pulp, without seeds. Together with the Tarocco and Sanguinello varieties, it is part of the so-called red or pigmented oranges. It is grown, in particular, in the eastern part of Sicily, in the territories around Etna, between the provinces of Catania, Enna, and Syracuse. It is mainly consumed squeezed, but thanks to its sweetness, it can also be used in the preparation of sweets and jams. The Arancia Bionda di Trebisacce, *C. sinensis* Biondo Tardivo of Trebisacce (CS-Italy), ecotype of the Ovale Calabrese cultivar, comes from Trebisacce, in the province of Cosenza. It is a late-ripening variety with long persistence of the fruit on the plant. The shape of the fruit is ovoid, with a light orange peel, and compact and juicy yellow-orange pulp. The ripening of this variety occurs in late spring [22]. This work aimed to identify chemical components, such as limonoids, polyphenols, and fatty acids, of Southern Italy citrus wastes to establish their biological potential. The antioxidant activity, registered in DPPH and ABTS in vitro assays, indicated a good antioxidant profile, while MTT cellular assay demonstrated a good ability of these phytocomplexes, derived from waste, to inhibit malignant proliferation, although at high doses. Moreover, principal component analysis revealed the correlations between chemical composition and biological activity.

## 2. Materials and Methods

### 2.1. Ultrasound-Assisted Extraction (UAE)

A total 20 g of pomaces (P) or peels (B) of Limone di Rocca Imperiale PGI (**LAMP**, **LAMB**), Arancia Rossa Moro (**MAMP**, **MAMB**), Arancia Bionda Tardivo (**BAMP**, **BAMB**), in 150 mL of absolute ethanol, was sonicated at 30 °C, 200 W, 40 KHz for 45 min. The suspensions were filtered using a Büchner funnel. The ethanol phases were then centrifuged at 8000 rpm for 10 min, and the supernatants concentrated to dryness at 30 °C. The dried samples were then weighted and stored at −20 °C until their analysis.

### 2.2. Gas Chromatography–Mass Spectrometry (GC-MS) Analyses

The analyses were carried out on an Agilent GC/MS system consisting of a 6890 N Network gas chromatograph and a 5973 Network Mass Selective Detector (5973N MSD) operating in 70-eV electron impact ionization mode. Mass spectra were acquired in full- scan mode in the range of 40–650 m/z. The GC was equipped with a 30-m HP-5MS capillary column (0.25-mm i.d., 0.25-mm film thickness), and helium was used as the carrier gas at a rate of 1 mL/min. The injector was set at 250 °C and was used in the split mode. The oven temperature program was set at an initial temperature of 70 °C (hold time 1.5 min), then a temperature ramp of 10 °C/min up to 280 °C was used to reach a final temperature of 280 °C (final hold time 20 min). The compounds were tentatively identified based on their Electron Ionization (EI) mass spectra using the NIST08 database.

### 2.3. Ultra-High-Performance Liquid Chromatography–High Resolution Mass Spectrometry (UHPLC-HRMS) Analyses

Each sample was dissolved in 1 mL of methanol/water (80:20 *v*/*v*). The samples were filtered through 0.45-μm membrane filter and analyzed by Liquid Chromatography–Mass Spectrometry. UHPLC-HRMS analyses were performed on a Shimadzu Nexera UHPLC system, consisting of a CBM-20A controller, two LC-30AD dual-plunger parallel-flow pumps, a DGU-20 AR5 degasser, an SPD-M20A photo diode array detector, a CTO-20A column oven, and an SIL-30AC autosampler. The system was coupled online to a hybrid Ion Trap-Time of Flight Mass Spectrometer (LCMS-IT-TOF, Shimadzu, Milan, Italy) equipped with an electrospray source ionization (ESI). For RP-UHPLC analyses, a Biphenyl 100 × 2.1 mm, 2.6 µm (L × I.D, particle size, Phenomenex^®^, Bologna, Italy) column was employed at a flow rate of 0.4 mL/min. The mobile phases consisted of (A) 0.1% CH_3_COOH in H_2_O and (B) ACN plus 0.1% CH_3_COOH. Analysis was performed in gradients as follows: 0–20.0 min, 2–20% B; 20.01–23.0 min, 20–60% B; 23.01–24.0 min, 60–99% B; 99% B hold for 1 min; returning to initial conditions in 0.1 min. The column oven was set to 40 °C, and 5 µL was injected. PDA detection parameters were the following: sampling rate 12 Hz, time constant 0.160 s, and chromatograms were extracted at 280 and 330 nm. LC data elaboration was performed by the LCMS solution^®^ software (Version 3.50.346, Shimadzu). MS detection was performed in negative and positive modes as follows: curve desolvation line (CDL), 250 °C; Block Heater, 250 °C; Nebulizing and Drying gas, 1.5 and 10 L/min; MS range, m/z 150–1500; ion accumulation time, 30 ms; ion trap repeat, 3. MS/MS was performed in data dependent acquisition (DDA), precursor ions selection was based on the base peak chromatogram (BPC) intensity of 700.000. Collision induced dissociation (CID), 50%; ion trap repeat, 1. The instrument was tuned using sodium trifluoroacetate (NaTFA). Metabolite annotation was based on accurate mass measurement, MS/MS fragmentation pattern, and comparison with in silico spectra with MS database searching. “Formula Predictor” software (Shimadzu) was used for the prediction of the molecular formula, using the following settings: maximum deviation from mass accuracy: 5 ppm, fragment ion information, and nitrogen rule.

### 2.4. 2,2′-Azino-Bis(3-Ethylbenzothiazoline-6-sulfonic Acid) Diammonium Salt (ABTS) Assay

The ABTS assay was performed as previously reported [23]. ABTS radical cations (ABTS^+^) were obtained by incubating, for 16 h at room temperature, 7 mM ABTS solution with 2.45 mM potassium persulfate solution. The solution, thus obtained, was diluted with ethanol until its absorbance (at 734 nM) reached a value of 0.70 ± 0.05. Increasing concentrations of the different extracts (1 to 200 µg/mL) were added to 1 mL of diluted ABTS^+^ solution, then the samples were mixed and incubated in darkness for 5 min. Ascorbic acid was used as positive control. For each sample, absorbance at 734 nM was measured using UV–vis spectrophotometer. The absorbance of each sample was related to the absorbance of the diluted ABTS^+^ solution without extracts, in percentage, (Abs_sample_/Abs_Control_ × 100), and plotted in order to calculate IC_50_ values.

### 2.5. 2,2′-Diphenyl-1-picrylhydrazyl (DPPH) Assay

The DPPH assay was carried out as previously described [23]. Briefly, increasing concentrations of the different extracts (1 to 200 µg/mL) were mixed with 33 µL of 1 mM DPPH methanol solution, and MeOH was added to make a final volume of 1 mL. Ascorbic acid was used as positive control. Mixtures were then vigorously stirred and incubated for 30 min in the dark, at room temperature. For each sample, the decrease in absorbance, at a wavelength of 517 nm, was evaluated and compared with respect to a control sample (without extracts), by using a UV–vis spectrophotometer (Ultrospec 2100 Pro, Amersham Biosciences/GE Healthcare, Amersham, UK). The absorbance of each sample was related to the absorbance of the DPPH mixture without extracts, in percentage, (Abssample/AbsControl × 100), and plotted in order to calculate IC_50_ values.

### 2.6. Cell Cultures

MCF-7 and MDA-MB-231 breast cancer cell lines and normal fibroblast BJ-hTERT cell line were purchased from the American Culture Collection (ATCC, Manassas, VA, USA). MCF-7 and MDA-MB-231 cells were cultured in DMEM/F12 (Sigma-Aldrich, St. Louis, MO, USA) supplemented with 1% penicillin/streptomycin (Sigma-Aldrich), 2 mM L-glutamine (Sigma-Aldrich), and 10% Fetal Bovine Serum (FBS, Sigma-Aldrich). BJ-hTERT cells were cultured in DMEM High-Glucose supplemented with 10% FBS, 2 mM L-glutamine, and 1% penicillin/streptomycin. All cells were cultured at 37 °C in a humidified atmosphere containing 5% CO_2_.

### 2.7. Cell Viability Assay

Cell viability was assessed using the 3-(4,5-Dimethyl-2-thiazolyl)-2,5-diphenyl-2H-tetrazolium bromide (MTT, Sigma-Aldrich) assay, as previously reported [24]. Briefly, MCF-7, MDA-MB-231, and BJ-hTERT cells were seeded in a 48-well plate (2 × 10^4^ cells/well), and treated for 72 h with different concentrations of the extracts (up to 400 µg/mL) in the above-mentioned media with reduced amount of FBS (2%). After treatment, the MTT solution was added to each well (to a concentration of 0.5 mg/mL), and plates were incubated for 2 h at 37 °C. DMSO was used to solubilize, in each well, the formed formazan crystals, which were then spectrophotometrically quantified at 570 nm, using a microplate reader (Synergy H1 microplate reader, BioTek, Winooski, VT, USA). The absorbance of each sample was related to the absorbance of the negative control, in percentage, (Abssample/AbsControl × 100).

### 2.8. Spectroscopic Profiling of Citrus via FTIR-ATR Analysis

A Spectrum Two Fourier transform infrared (FTIR) spectrometer (Perkin Elmer Italia Spa, Milan, Italy), equipped with an attenuated total reflection (ATR) accessory incorporating a top plate, fitted with a ZnSe crystal, was used to acquire the infrared spectra of citrus extract samples. The lab air was used as background FTIR-ATR spectrum to check the cleanliness, the instrumental conditions, and the room environmental interference due to H_2_O and CO_2_. IR sample spectra, laid on the ATR top without any pre-treatment, were recorded in triplicate in the wavenumber range from 4000 to 450 cm^−1^. Sample spectra were acquired by taking the average spectrum of 64 scans with a resolution of 4 cm^−1^. IR data were arranged in an 18 × 3551 matrix and subjected to multivariate analysis [25]. A preliminary selection of the wavenumber range was essential before data analysis, and data matrix was resized by keeping out the spectral region 4000–1850 cm^−1^. The spectral window 4000–2500 cm^−1^ was characterized by broad signals associated with the stretching vibrations of hydroxyl groups and aromatic C-H that were poor in information about phenolic compounds. The range between 2500 and 1850 cm^−1^ did not contain spectral relevant information, aside from the CO_2_ bands. Therefore, the remaining range of 1850–450 cm^−1^ (18 × 1401) was used for the multivariate data analysis [26].

### 2.9. Multivariate Data Analysis

Principal component analysis (PCA) is an exploratory technique and allows the reduction in data dimensionality by extracting the information available in the spectra, projecting the samples and variables on the new variables called principal components (PCs) [27]. PCA was performed in order to evaluate the metabolic fingerprint in terms of phenolic compounds from the different citrus extracts; it was carried out using the Singular Value Decomposition algorithm (SVD). The Unscrambler X software version 10.6 from CAMO (Computer-Aided Modelling, Trondheim, Norway) was used for the multivariate elaboration of the experimental data [28].

### 2.10. Statistical Analysis

Non-linear regression analysis was used to calculate IC_50_ values, by generating dose–response curves. Analysis of variance (ANOVA) was applied on data from at least three replicate determinations, by using GraphPad Prism 8 software (GraphPad Software, San Diego, CA 92108, USA) and a *p* value ≤ 0.05 was considered statistically significant.

## 3. Results and Discussion

### 3.1. Extraction of Bioactive Compounds via UAE

The extraction yields of all the matrices are reported in Table 1.

### 3.2. Chemical Characterization of the Extracts

The obtained extracts were analyzed through GC-MS analysis and UHPLC-HRMS analysis. Among the pomace extracts, **LAMP** was shown to be rich in volatile compounds, including 5-methyl-2-furancarboxaldehyde, squalene, and different fatty acids and derivatives as reported in Table 2. **MAMP** presents only some fatty acid derivatives, while **BAMP** also showed traces of benzoic acid (Table 2). Peel extracts showed a similar composition including fatty acid derivatives, quinic acid (**LAMB**), and inositols (**MAMB**) as reported in Table 3.

According to UHPLC-HRMS analysis, all the extracts contained similar structural compounds. In particular, limonin-hexoside, neodiosmin, obacunone glucoside, and deacetyl nomilinic acid glucoside were identified as limonoid structures present in all the samples, as well as different polyphenols including naringenin-glucoside, hesperetin-*O*-hexoside-*O*-rhamnoside-*O*-glucoside, diferuloyl-glucaric acid ester, and chlorogenic acid (Table 4). Surprisingly, **BAMP** and **BAMB** extracts did not contain flavonoid structures, such as quercetin and rutin derivatives. Of note, some methoxyflavones were identified in the samples, including tangeretin and sinensetin, which were absent in **LAMB** and **LAMP** (Table 5).

### 3.3. Biological Evaluation of the Extracts

#### 3.3.1. Antioxidant Profile of Extracts from Citrus Fruits Wastes

**Figure 1:**
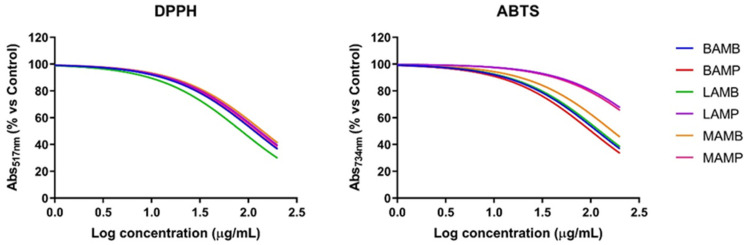
Antioxidant profile of extracts from citrus fruits wastes.

The antioxidant potential of different extracts was evaluated by DPPH and ABTS assays using ascorbic acid as positive control (Figure 1, Table 6). The DPPH assay showed comparable antioxidant profiles between the different extracts, characterized by IC_50_ values between 115.4 and 140.6 µg/mL, except for **LAMB**, whose antioxidant profile, in this assay, turned out to be slightly higher, with an IC_50_ value of 85.18 µg/mL. On the other hand, the results obtained by the ABTS assay confirm a fair and very similar antioxidant potential of the different extracts, with IC_50_ values between 100.8 and 167.8 µg/mL, except for **LAMP** and **MAMP**, which provided higher IC_50_ values (417.2 and 381.0 µg/mL, respectively). Overall, the obtained results are consistent with the composition of the tested extracts, which displayed a similar phenolics composition.

#### 3.3.2. Extracts from Citrus Fruits Wastes Exert Antiproliferative Effects into Breast Cancer Cells

**Figure 2:**
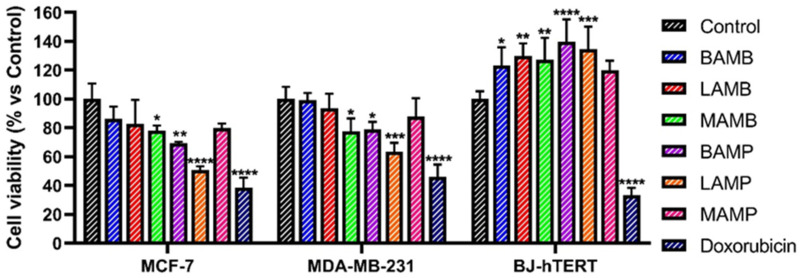
Extracts from citrus fruits wastes exert antiproliferative effects in breast cancer cells. Cells were treated for 72 h with the different extracts (400 µg/mL) and tested by MTT assay. Doxorubicin (1 µg/mL) was used as positive control. Results are expressed as percentage of cell viability versus control. Values represent mean ± S.D. of three independent experiments, each one performed with triplicate samples. * *p* < 0.05; ** *p* < 0.01; *** *p* < 0.005; **** *p* < 0.001.

To better investigate the biological properties of the different extracts, their effects on the proliferation of breast cancer cells were evaluated. In order to take into account breast cancer diversity, in this study, two different tumor cell lines were used: the luminal MCF-7 tumor line and the triple negative tumor line MDA-MB-231, a tumor subtype characterized by high aggressiveness and invasiveness. Cells were treated for 72 h with different concentrations of the extracts (up to 400 µg/mL), then their proliferation was evaluated by MTT assay and compared to that of the untreated control cells. The obtained results highlighted the ability of the tested extracts to exert antiproliferative effects on cancer cells just at the highest concentration used (400 µg/mL), (Figure 2). In particular, **MAMB**, **BAMP,** and **LAMP** were able to statistically significantly reduce the proliferation/viability of both tumor lines used and, among all the extracts, the greatest inhibitory effects were observed after **LAMP** treatment. To evaluate the selectivity toward tumor cells of the observed effects, the extracts were also tested on non-tumorigenic human fibroblast cell line BJ-hTERT. Notably, the treatment with citrus fruit wastes extracts did not determine any inhibitory effect on cell proliferation/viability but, on the contrary, it would seem to induce significant cytoprotective effects toward BJ-hTERT cells. This biological behavior considerably differs from that of doxorubicin, used as a positive control in this study, which showed a stronger cytotoxic activity but does not discriminate the nature of the cells.

#### 3.3.3. Principal Component Analysis (PCA) to Infrared Fingerprint Characterization

**Figure 3:**
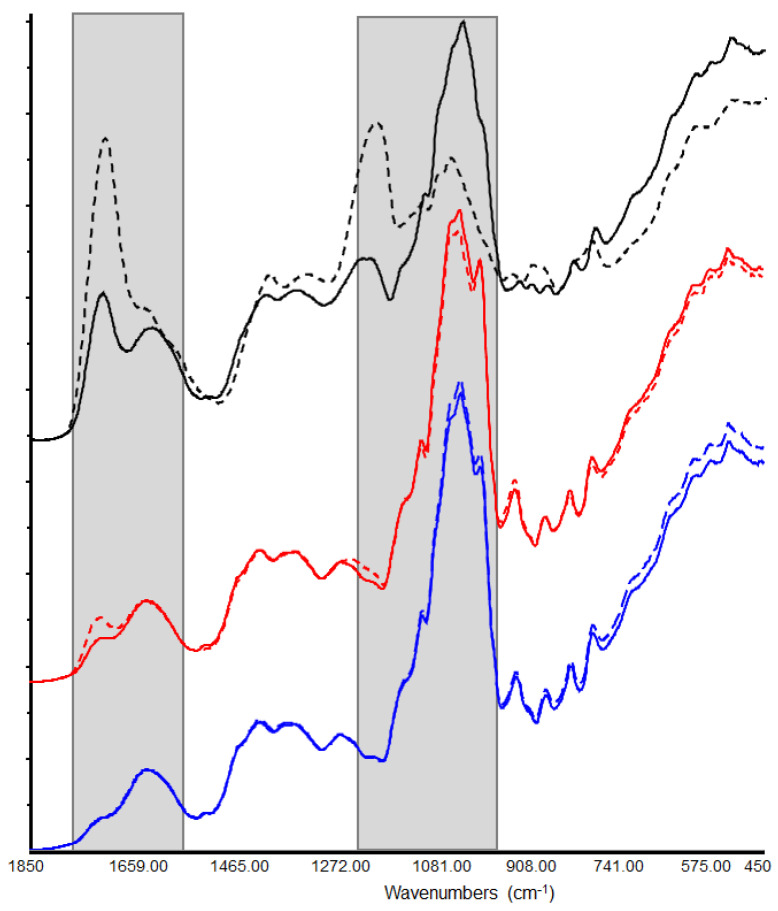
FTIR spectra for the different citrus samples: **BAMB** (blue line), **BAMP** (dotted blue line), **MAMB** (red line), **MAMP** (dotted red line), **LAMB** (black line), **LAMP** (dotted black line).

**Figure 4:**
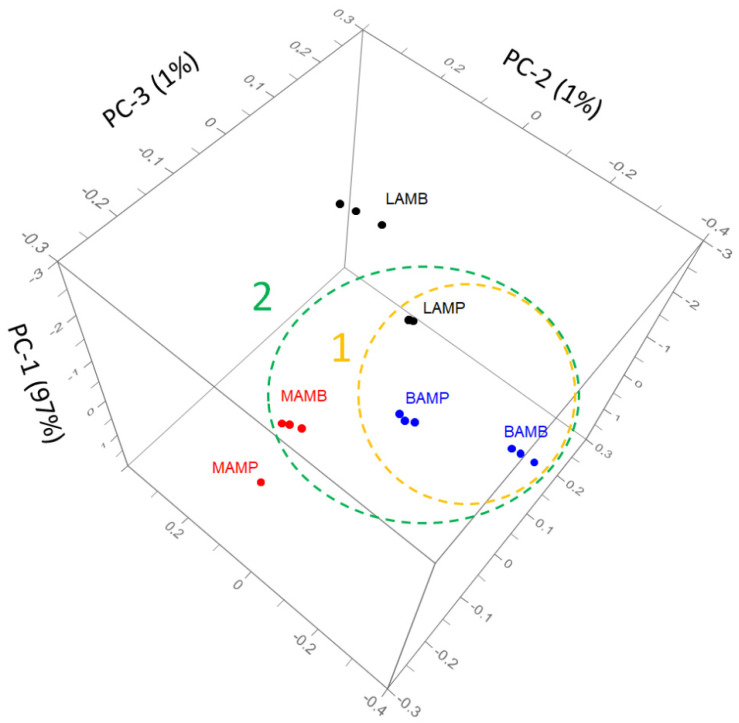
Three-dimensional score plot PC1-3 obtained in PCA modelling to FTIR data from citrus extracts in the range 1800–450 cm^−1^. Yellow cluster (1) referred to citrus samples with higher antioxidant activity (ABTS and DPPH); green cluster (2) referred to higher antiproliferative activity (MTT).

Figure 3 shows the citrus spectra recorded from the extraction procedure. The common spectral bands 1750–1500, 1450–1200, and 1199–700 cm^−1^ are associated with different classes of polyphenols and their major groups. The phenolic compounds have a large and overlapped infrared fingerprint and using single peaks or limited wavenumber ranges to distinguish for the different samples is very difficult. In this case, the application of chemometric tools can be useful to interpret data patterns and for sample discrimination [27]. PCA elaboration was applied to the spectral data matrix, with the aim of discriminating the different polyphenols contribution of citrus samples. Figure 4 describes the 3D scores plot by considering first, second, and third principal components with a total % explained variance of 99%.

The sample grouping was evident, and group discrimination was possible to distinguish the samples according to the fruits and portion treated, confirming the differences in phenols composition evaluated by chromatographic study. In the 3D score plot, a second grouping approach can be observed, where the samples were grouped considering similar antioxidant or antiproliferative activities (Figure 4). Most likely, group discrimination was due to the different distribution of the extract polyphenols composition. By comparing the X-loading values calculated for the elaborated waves with respect to the first three PCs, some characteristic citrus bands were highlighted. PCA showed a relative abundance of information in the wave ranges 1100–950 and 1800–1700 cm^−1^ (highlighted in Figure 3). The first spectral window is characteristic of the flavonoid content of the extracts. It has been noted that some flavonoids are linked to carbohydrates and that the spectral region between 1200 and 950 cm^−1^ contains functional groups mainly from carbohydrates [26]. The second spectral window can still be referred to the polyphenol content; moreover, in this range, the characteristic peaks of limonin, neodiosmin, and obacunone fingerprints are also detectable [29,30,31]. The LAMP sample had predominant bands in both windows, which may explain the highest relative antioxidant and antiproliferative activities.

## 4. Conclusions

This work evaluated, for the first time, the chemical composition of the wasted part (peel and pomace) of autochthonous citrus fruits derived from two main citrus producing areas, Calabria and Sicily, in South Italy. The antioxidant and antiproliferative activities of six extracts, namely **LAMB**, **LAMP, BAMB**, **BAMP**, **MAMB**, and **MAMP**, were investigated. **LAMP** and **MAMP** showed an excellent antioxidant activity in DPPH and ABTS radical-scavenging assays, while the greatest antiproliferative effect on MCF-7 and MDA-MB-231 breast cancer cell lines was detected after **LAMP** treatment at 400 μg/mL. Overall, this study highlights the importance of an appropriate recycling of citrus fruit wastes native to the study area, as their hydroalcoholic extracts could open the way for the development of new nutraceuticals, useful as adjuvants for prevention and treatment of many free-radical-associated diseases.

## Figures and Tables

**Table 1:** Extraction yields of pomace and peel of Limone di Rocca Imperiale, Arancia Rossa Moro, Arancia Bionda Tardivo.

Citrus *cv.*	Pomace	Yield (g)	Peel	Yield (%)
Limone di Rocca Imperiale	**LAMP**	36%	**LAMB**	85%
Arancia Rossa Moro	**MAMP**	25%	**MAMB**	79%
Arancia Biondo Tardivo	**BAMP**	46%	**BAMB**	42%

**Table 2:** Qualitative GC-MS analysis of LAMP, MAMP, and BAMP extracts.

RT	Analyte	LAMP	MAMP	BAMP
4.23	2,5-Furandione, 3-methyl-	+	-	-
4.39	2-Furancarboxaldehyde, 5-methyl-	+	-	-
5.24	1-Hexanol, 2-ethyl-	+	-	-
6.02	4H-Pyran-4-one, 3-hydroxy-2-methyl (Maltol)	+	-	-
7.02	4H-Pyran-4-one, 2,3-dihydro-3,5-dihydroxy-6-methyl-	+	-	+
7.55	Benzoic acid	-	-	+
8.17	Benzofuran, 2,3-dihydro-	-	-	+
8.33	2-Furancarboxaldehyde, 5-(hydroxymethyl)-	+	+	+
12.70	2,6-Dimethyl-3-(methoxymethyl)-*p*-benzoquinone	-	-	+
15.70	Tetradecanoic acid (Miristic acid)	+	-	+
15.95	1,2-Benzenedicarboxylic acid	+	-	-
16.00	1-Hexadecene	+	-	-
16.75	Hexadecanoic acid (Palmitic acid)	+	-	+
17.98	1-Octadecene	+	-	-
18.24	Heptadecanoic acid	-	+	-
18.56	9,12-Octadecadienoic acid (Linoleic acid)	+	-	+
18.90	Octadecanoic acid, ethyl ester	-	+	-
19.70	Tetradecane	+	-	-
20.61	Octadecanoic acid, 2-methylpropyl ester	-	+	-
21.40	9,12-Octadienal	-	-	+
21.64	Hexadecanoic acid, 2,3-dihydroxy-	+	-	+
23.32	Octadecanoic acid, 2-hydroxy-	+	-	+
24.29	Squalene	+	-	-

(+) presence; (-) absence.

**Table 3:** Qualitative GC-MS analysis of LAMB, MAMB, and BAMB extracts.

RT	Analyte	LAMB	MAMB	BAMB
4.11	2,5-Furandione, 3-methyl-	+	-	-
4.39	2-Furancarboxaldehyde, 5-methyl-	+	-	-
5.23	1-Hexanol, 2-ethyl-	+	-	-
6.06	2,4(1H,3H)-Pyrimidinedione, 5-methyl-	+	+	+
6.14	2-Furancarboxylic acid, methyl ester	+	-	-
7.10	4H-Pyran-4-one, 2,3-dihydro-3,5-dihydroxy-6-methyl-	+	+	+
7.36	Benzoic acid	-	+	-
8.22	2-Furancarboxaldehyde, 5-(hydroxymethyl)-	+	+	+
9.43	Ethanone, 1-(2-hydroxy-5-methyl)-	+	-	-
11.42	Decanoic acid	+	-	-
12.63	2,6-Dimethyl-3-(methoxymethyl)-*p*-benzoquinone	+	-	+
12.67	1,4-Dihydrophenanthrene	-	+	-
13.37	Quinic acid	+	-	-
13.83	Cyclododecane	+	-	-
13.93	Octanoic acid	-	+	-
15.69	Tetradecanoic acid (Miristic acid)	-	-	+
16.00	1-Tetradecene	+	-	-
17.61	Inositols	-	+	-
17.76	9-Octadecen-1-ol	+	-	-
17.97	1-Octadecene	+	-	-
18.93	9,12-Octadecadienoic acid (Linoleic acid)	+	-	+
20.27	Octadecanoic acid, 2-methylpropyl ester	+	-	-
21.64	Hexadecanoic acid, 2,3-dihydroxy-	+	+	+
23.26	Octadecanoic acid, 2-hydroxy-	+	+	-
23.38	Octadecanoic acid (Stearic acid)	-	+	-

(+) presence; (-) absence.

**Table 4:** Qualitative profile of phytochemicals isolated in citrus extracts via UHPLC-HRMS.

Peak	r_t_	Compound	[M-H]^-^	MS/MS	Molecular Formula	Error (ppm)	BAMB	BAMP	MAMB	MAMP	LAMB	LAMP
1	7.57	Ferulic acid-glc	355.1049	193.0498	C_16_H_20_O_9_	4.50	Yes	Yes	Yes	Yes	Yes	Yes
2	8.29	Synapoyl glucose	385.1152	205.0509; 190.0285	C_17_H_22_O_10_	2.86	Yes	Yes	Yes	Yes	Yes	Yes
3	8.89	Vicenin II	593.1488	353.0683; 383.0770; 297.0777	C_27_H_30_O_15_	2.30	Yes	Yes	Yes	Yes	Yes	Yes
4	9.82	Diosmetin 6.8-di-C-glc	623.1613	383.0778; 413.0846	C_28_H_32_O_16_	2.70	Yes	Yes	Yes	Yes	Yes	Yes
5	10.87	Luteolin-*O*-hex.-*O*-rham-*O*-glc	757.2175	287.0559; 449.1068	C_30_H_50_O_25_	−2.53	Yes	Yes	Yes	Yes	Yes	Yes
6	11.12	Naringenin-glc	433.1121	271.0604	C_21_H_22_O_10_	2.55	Yes	Yes	Yes	Yes	No	No
7	11.45	Limonin-hex	649.2480	443.2060; 605.2650	C_32_H_42_O_14_	3.61	Yes	Yes	Yes	Yes	Yes	Yes
8	12.44	Luteolin-*O*-rhamn.-*O*-glc	595.1628	287.0652	C_27_H_30_O_15_	5.30	Yes	Yes	Yes	Yes	Yes	Yes
9	12.70	Poncirin	593.1481	285.0383	C_28_H_32_O_14_	−3.31	Yes	Yes	Yes	Yes	Yes	Yes
10	12.95	Hesperetin-*O*-hex-*O*-rham-*O*-glc	771.2338	301.0712; 463.1206; 609.1755	C_34_H_44_O_20_	−1.94	Yes	Yes	Yes	Yes	No	No
11	13.98	Deacetyl nomilin glc	651.2643	653.2657; 591.2410	C_32_H_44_O_14_	2.23	Yes	Yes	Yes	Yes	Yes	Yes
12	14.21	Nomilic acid hex	711.2843	607.2746; 651.2593	C_34_H_48_O_16_	3.1	Yes	Yes	Yes	Yes	Yes	Yes
13	14.31	Narirutin	579.1691	271.0664	C_27_H_32_O_14_	2.42	Yes	Yes	Yes	Yes	Yes	Yes
14	14.85	Hesperetin-*O*-hex-rham-*O*-glc-*O*-hex	915.2771	301.0719; 463.1208; 609.1783; 771.2310	C_40_H_52_O_24_	−2.73	Yes	Yes	Yes	Yes	No	No
15	14.90	Diferuloyl-glucaric acid ester	561.1209	367.0659	C_26_H_26_O_14_	−4.1	Yes	Yes	Yes	Yes	No	No
16	15.15	Neodiosmin	607.1638	299.0562	C_28_H_32_O_15_	−1.70	Yes	Yes	Yes	Yes	Yes	No
17	15.83	Hesperetin-*O*-rham-*O*-glc (Hesperidin)	609.1819	301.0763	C_28_H_34_O_15_	2.71	Yes	Yes	Yes	Yes	Yes	Yes
18	17.16	Obacunone glc	633.2556	427.2130; 359.1840; 331.1891	C_32_H_42_O_13_	−4.58	Yes	Yes	Yes	Yes	Yes	Yes
19	20.07	Dydimin	593.1845	285.0777	C_28_H_34_O_14_	2.05	Yes	Yes	Yes	Yes	Yes	Yes
20	1.02	Chlorogenic acid	353.0874	173.0489; 191.0576	C_16_H_18_O_9_	−1.13	No	No	Yes	Yes	Yes	Yes
21	4.82	3′- Coumaroylquinic acid	337.0945	163.0417 119.0558	C_16_H_18_O_8_	4.75	No	No	Yes	Yes	Yes	No
22	5.25	4′-Coumaroylquinic acid	337.0952	173.0458; 163.0418	C_16_H_18_O_8_	2.30	No	No	Yes	Yes	Yes	No
23	6.94	Feruloylquinic acid	367.1053	193.0531; 134.0390	C_17_H_20_O_9_	4.90	No	No	Yes	Yes	Yes	No
24	10.65	Diosmetin 6.8-di-C-hex	623.1613	383.0778; 413.0846	C_28_H_32_O_16_	2.70	No	No	Yes	Yes	Yes	Yes
25	11.08	Deacetyl nomilinic acid glc	669.2746	609.2511	C_32_H_46_O_15_	−2.69	No	No	Yes	Yes	Yes	Yes
26	11.32	Rutin	609.1434	301.0333; 271.0248; 255.0277	C_27_H_30_O_16_	−4.43	No	No	No	No	Yes	Yes
27	11.70	Quercetin-3-*O*-hex	463.0849	301.0347; 271.0248; 255.0277	C_21_H_20_O_12_	−3.25	No	No	Yes	Yes	Yes	No
28	13.27	Diosmetin-*O*-hex	461.1088	298.0505; 341.0663	C_22_H_22_O_11_	−0.43	No	No	Yes	Yes	Yes	Yes
29	13.56	Diosmetin-*O*-hex	461.1088	298.0505; 341.0663	C_22_H_22_O_11_	−0.43	No	No	No	No	Yes	Yes
30	7.30	Quercetin-*O*-hex-*O*-rham-*O*-glc	771.1995	301.0359;	C_33_H_40_O_21_	0.78	No	No	No	Yes	No	Yes

Abbreviations: glc, glucoside; hex, hexoside; rham, rhamnoside.

**Table 5:** Qualitative profile of methoxyflavones isolated in citrus extracts via UHPLC-HRMS.

Peak	r_t_	Compound	[M+H]^+^	MS/MS	Molecular Formula	Error (ppm)	BAMB	BAMP	MAMB	MAMP	LAMB	LAMP
1	22.88	Sinensetin isomer	373.1191	312.0885; 329.0911	C_20_H_20_O_7_	0.95	Yes	Yes	Yes	Yes	No	No
2	23.16	Sinensetin	373.1191	312.0885; 329.0911	C_20_H_20_O_7_	0.95	Yes	Yes	Yes	Yes	No	Yes
3	23.37	Hexamethoxyflavone	403.1299	373.0823	C_21_H_22_O_8_	−2.8	Yes	Yes	Yes	Yes	No	No
4	23.51	Tetramethoxyflavone	343.1082	282.0779	C_19_H_18_O_6_	−3.30	Yes	Yes	Yes	Yes	Yes	No
5	23.75	Tangeretin	373.1187	343.0740	C_20_H_20_O_7_	−4.30	Yes	Yes	Yes	Yes	No	No

**Table 6:** Antioxidant activity (IC_50_ values) of pomace and peel of Limone di Rocca Imperiale, Arancia Rossa Moro, and Arancia Biondo Tardivo.

DPPH						
Citrus *cv.*	pomace	IC_50_ (µg/mL)	SD	peel	IC_50_ (µg/mL)	SD
Limone di Rocca Imperiale	LAMP	130.3	30.78	LAMB	85.18	11.65
Arancia Rossa Moro	MAMP	125.8	18.09	MAMB	140.6	28.26
Arancia Biondo Tardivo	BAMP	117.0	22.15	BAMB	115.4	24.25
Ascorbic acid (positive control)		6.3	1.5			
		**ABTS**				
Citrus *cv.*	pomace	IC_50_ (µg/mL)	SD	peel	IC_50_ (µg/mL)	SD
Limone di Rocca Imperiale	LAMP	417.2	26.43	LAMB	124.6	32.04
Arancia Rossa Moro	MAMP	381.0	34.43	MAMB	167.8	21.38
Arancia Biondo Tardivo	BAMP	100.8	17.66	BAMB	116.5	16.13
Ascorbic acid (positive control)		2.2	0.8			

IC_50_ values and standard deviation (SD) were calculated by non-linear regression analysis of data from three independent experiments.

## Data Availability

All of the data is contained within the article.
